# Neuropathological evaluation of a vertebrate brain aged ~ 245 years

**DOI:** 10.1007/s00401-020-02237-4

**Published:** 2020-10-16

**Authors:** Daniel Erny, Klara B. Jakobsdóttir, Marco Prinz

**Affiliations:** 1 Faculty of Medicine, Institute of Neuropathology, University of Freiburg, Breisacher Str. 64, 79106 Freiburg, Germany; 2 Demersal Division, Marine and Freshwater Research Institute, Hafnarfjörður, Iceland; 3 Signalling Research Centres BIOSS and CIBSS, University of Freiburg, Freiburg, Germany; 4 Faculty of Medicine, Center for Basics in NeuroModulation (NeuroModulBasics), University of Freiburg, Freiburg, Germany

Aging of the human and primate brain is associated with a wide range of distinct alterations affecting cell physiology, tissue integrity and architecture of the central nervous system (CNS) [15]. The limited self-renewal capacity of postmitotic neurons through adult neurogenesis [12] renders these key cells more susceptible to various exogenous and endogenous threats such as toxic agents, pathophysiological conditions throughout a life time. Similarly, non-neuronal cells such as microglia and other CNS-associated macrophages have been proven to be relatively long-lived in human and rodent brains with relatively low rates of homeostatic proliferation [8, 16, 21] making them equally susceptible to environmental stimuli.

A number of changes observed in aged human brains are attributed to increased levels of local oxidative stress during senescence [7]. Furthermore, several intra- or extracellular depositions, i.e. lipofuscin, typically accumulate with age [9]. Most age-related neurodegenerative diseases, such as Alzheimer’s or Parkinson’s disease exhibit potential toxic aggregations as histopathological hallmarks (e.g. extracellular amyloid beta or intraneuronal alpha-synuclein), resulting in loss of neurons in defined anatomical regions.

To gain further insight into the mechanisms of aging, we identified an exceptionally aged brain of a Greenland shark (*Somniosus microcephalus*) as the perfect candidate. These rare vertebrates are known for their extreme longevity of up to 392 ± 150 years of life expectancy accompanied by a relatively isolated hermit-like life style preserving their CNS from desperate environmental stress factors [11, 14]. We report for the first time the structural features of a ~ 245 ± 38 years old brain from a female Greenland shark that was caught with bottom trawl deep off West Iceland (62.39.32°N and 24.39.31°W at depth of 722 m) during an annual autumn survey of the Icelandic Marine and Freshwater Institute in November 2017. The age of the Greenland shark was estimated according to the correlating body length of 460 cm [14].

Macroscopically, the CNS was symmetrically structured and exhibited no obvious pathological alterations (Fig. 1a). At a histological level, the brain surfaces of the telencephalon were covered by normal appearing leptomeninges (Fig. 1b). Within deeper cortical layers next to smaller cells, presumably presenting neurons, larger triangle-shaped neurons with a size of around 50 µm were observed that mimicked morphologically pyramidal neurons known from humans (Fig. 1c). Further, regularly interspersed smaller round nuclei (10–15 µm) with dense chromatin were detected which could be attributed to oligodendrocytes. In contrast, microglia are known to present more elongated bean-shaped nuclei with a typical heterochromatin pattern [10]. These typical microglia-like cells were easily detectable in the sections and were characteristically located in a close proximity to neurons, as known from the human brain (Fig. 1c). Just recently, microglia have been systematically examined and genetically profiled in several species but sharks were not included in this study [6]. However, this cross-species comparison highlighted several evolutionary conserved and divergent transcriptional features of microglia [6]. Notably, within the Virchow-Robin spaces numerous cells with thin and elongated nuclei with typical heterochromatin pattern were found, potentially resembling perivascular macrophages as described in other species (Fig. 1g).

**Fig. 1:**
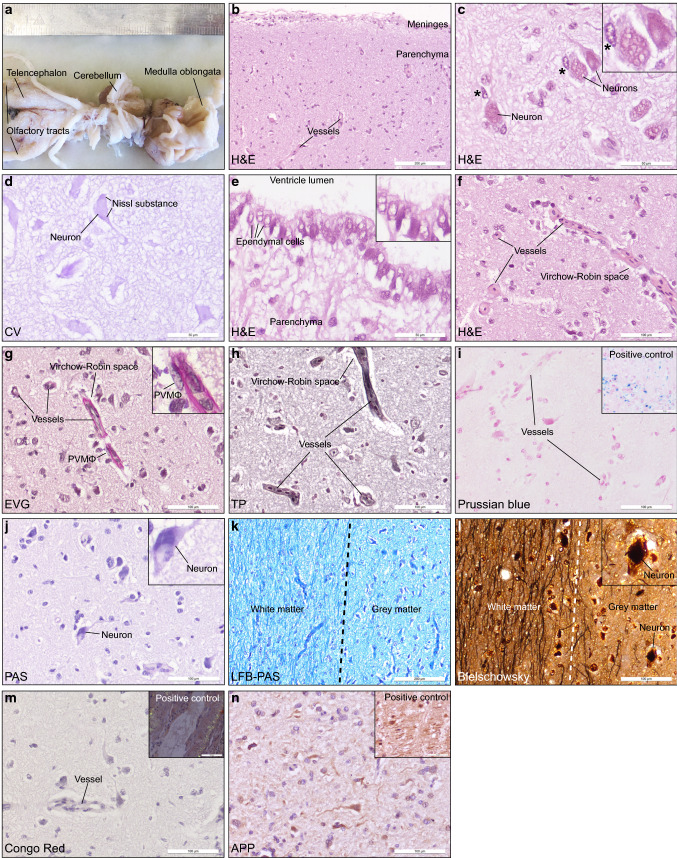
Macroscopic and histomorphological findings of a ~ 245 years old CNS. **a** Macroscopic few on an approximately 245 years aged Greenland shark brain. **b** Haematoxylin & eosin (H&E)—stained histological specimen of the telencephalon. Scale bar: 200 µm. **c** H&E-stained slide. Putative microglial cells with bean-shaped nucleus and typical heterochromatin pattern are marked with asterisk (*). Insert: Neurons and microglial cell (asterisk). Scale bar: 50 µm. **d** Cresyl violet (CV)—stained section. Scale bar: 50 µm. **e** H&E-stained specimen. Insert: Magnification of ependymal layer. Scale bar: 50 µm. **f** H&E—stained tissue sample. Scale bar: 100 µm. **g** Elastica-van-Gieson (EVG)—stained section. Vessels, Virchow-Robin space and putative perivascular macrophage (PVMɸ) are indicated. Insert: Vessel with PVMɸ. Size bar: 100 µm. **h** Tibor PAP (TP) silver—stained section. Scale bar: 100 µm. **i** Prussian blue-staining. Scale bar: 100 µm. Insert: positive control. Scale bar: 50 µm. **j** PAS-stained section. Scale bar: 100 µm. **k** LFB-PAS—stained tissue specimen. Scale bar: 200 µm. **l** Bielschowsky silver–staining. Scale bar: 100 µm. **m** Congo red—staining. Scale bar: 100 µm. Insert: positive control. Scale bar: 100 µm. **n** Immunohistochemistry for APP. Scale bar: 100 µm. Insert: scale bar: 50 µm

Importantly, virtually all age-related changes commonly found in elderly human brains, such as protein depositions, vascular or parenchymal calcifications were completely absent in the Greenland shark CNS. Furthermore, classical signs of neurodegeneration were not identifiable in various telencephalic regions (Fig. 1) and in the medial pallidum (Suppl. Figure 1), suggesting that the brain of vertebrates can be morphologically preserved for an extremely long time as it was described for human centenarians [13, 20].

We speculate that this finding might be caused by the specific environmental factors of this animal. Greenland sharks are reported to live predominantly in 4°C cold waters in the Arctic deep sea with remarkably slow movements [2] with potential low aerobic metabolism and little mitochondrial oxidative stress as well as and high concentrations of e.g. trimethylamine that might be neuroprotective. It is further suggested that Greenland sharks feature a relatively low blood pressure compared to other sharks [18] that might reduce the risk of hypertension-related CNS damage such as stroke or cognitive decline [5]. Up to now, information about genetics from Greenland sharks are largely missing [4, 17]. Further, we investigated the CNS of three additional chondrichthyan species that were caught in the same habitat: *Amblyraja radiata* (thorny skate), *Centroscyllium fabricii* (black dogfish) and *Chimaera monstrosa* (rabbit fish**)**. There were no macro- and microscopical lesions detectable, although the known life expectancy is rather short compared to Greenland sharks (Suppl. Figures 2, 3) [1, 3, 14, 19].

In summary, our study suggests that the CNS integrity can be preserved in vertebrates for centuries, while the precise impact of intrinsic and environmental factors for the process of brain aging will need to be determined in future studies.

## Electronic supplementary material

Below is the link to the electronic supplementary material.

Supplementary material 1 (PDF 5398 kb)

Supplementary material 2 (PDF 2334 kb)

Supplementary material 3 (PDF 368 kb)
