# Supplementary figures and images for: Neuropathological evaluation of a vertebrate brain aged ~ 245 years
Source: Acta Neuropathol. 2020 Oct 16;141(1):133–6. doi: 10.1007/s00401-020-02237-4 (PMC7785537; doi:10.1007/s00401-020-02237-4)

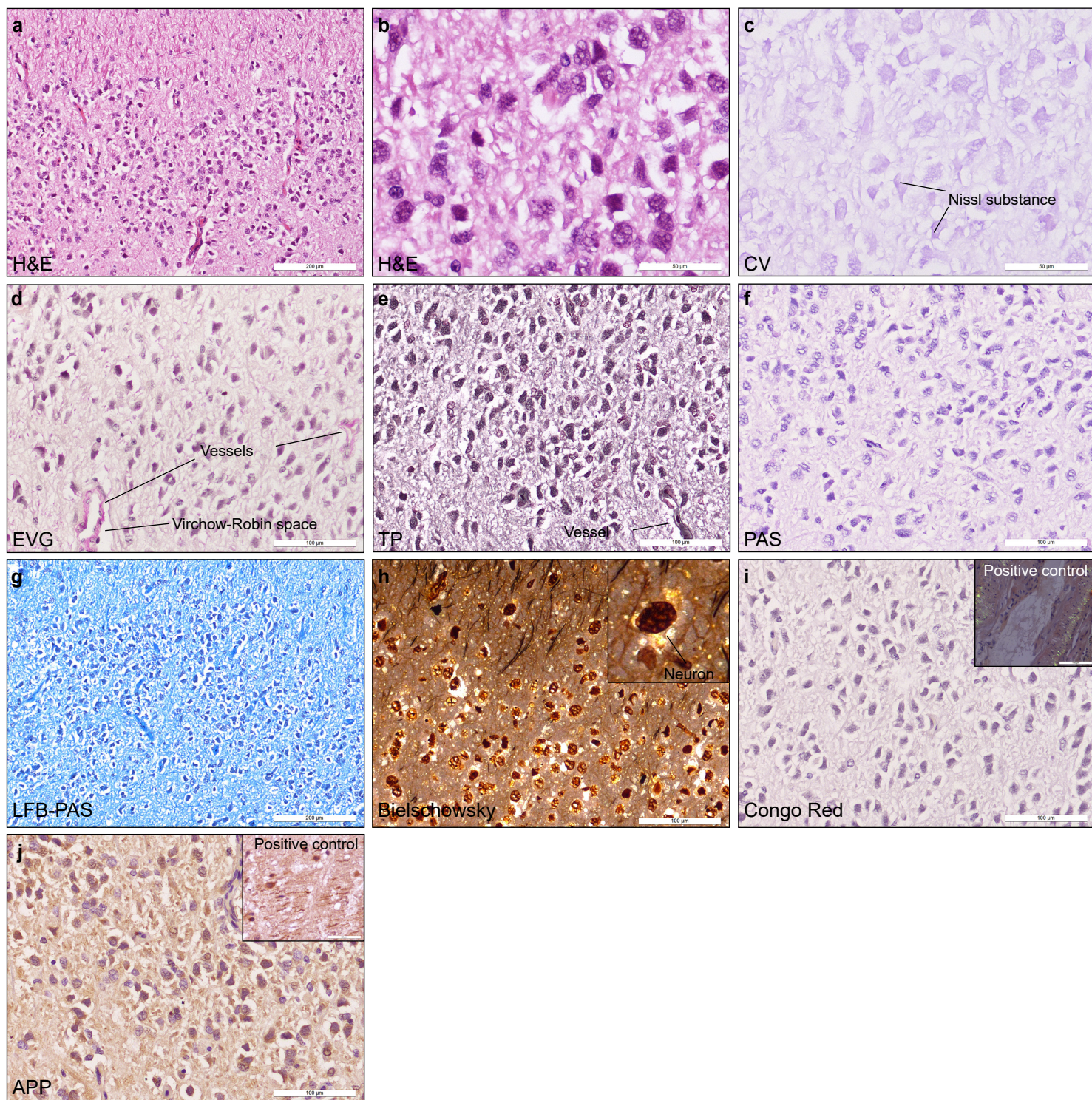

**Supplementary Figure 1**

Supplement: Supplementary file 1 — Supplementary material 1 (PDF 5398 kb) [file 401_2020_2237_MOESM1_ESM.pdf]

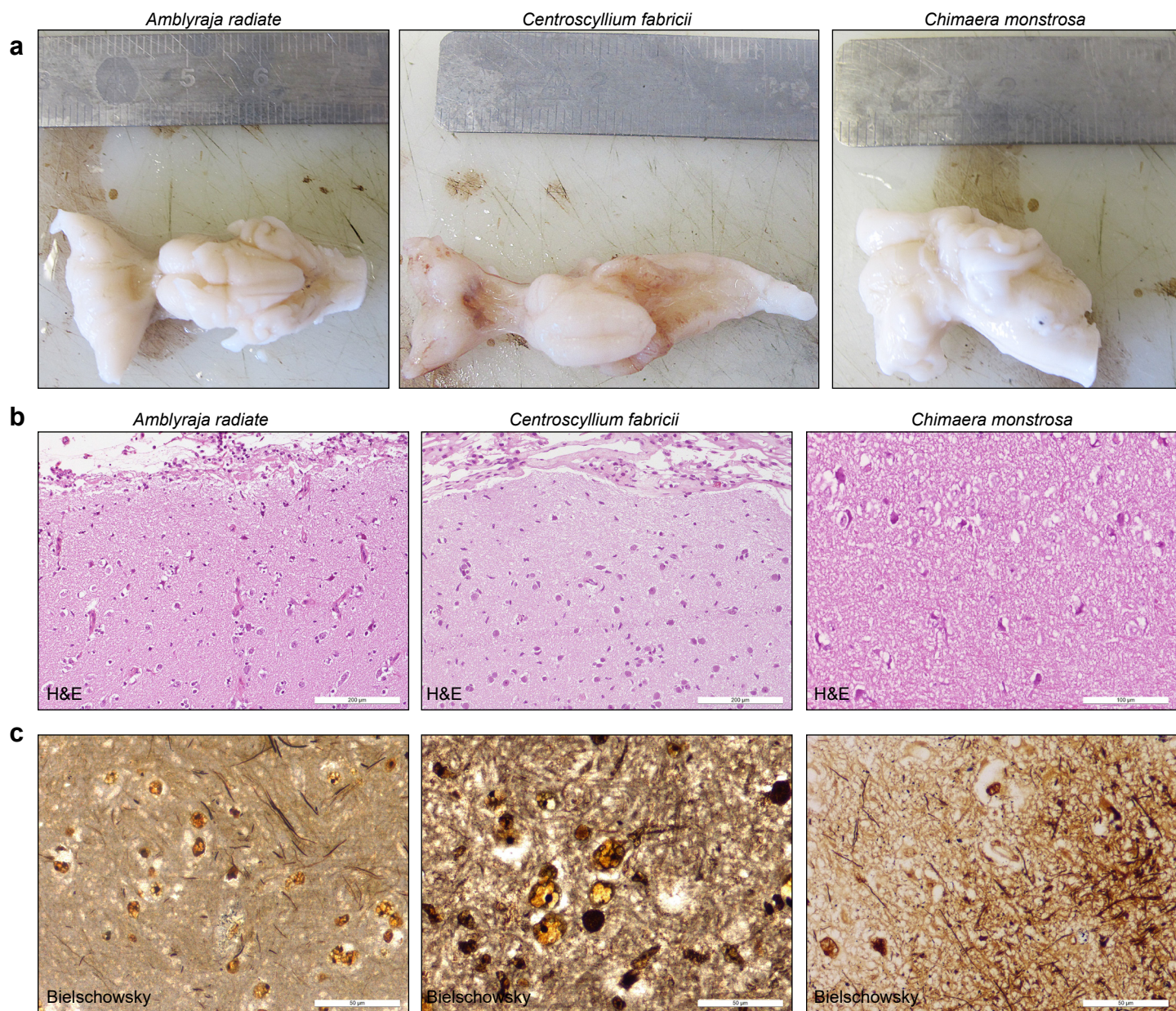

**Supplementary Figure 2**

Supplement: Supplementary file 2 — Supplementary material 2 (PDF 2334 kb) [file 401_2020_2237_MOESM2_ESM.pdf]

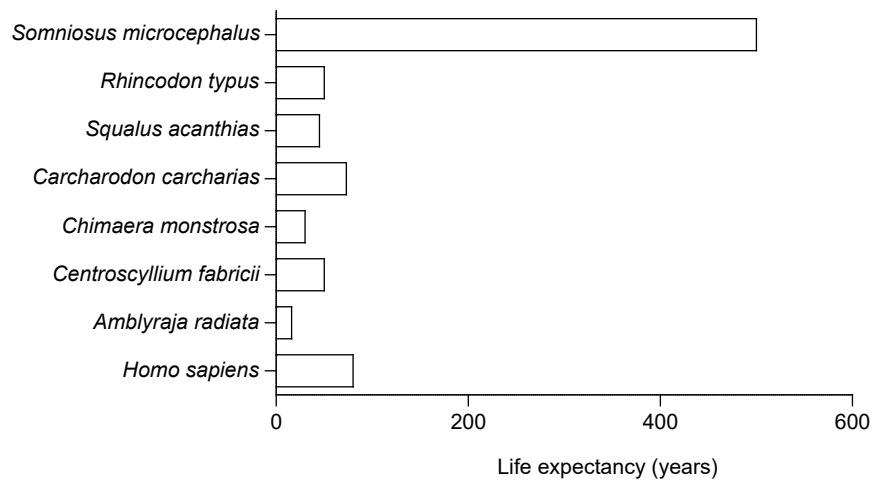

**Supplementary Figure 3**

Supplement: Supplementary file 3 — Supplementary material 3 (PDF 368 kb) [file 401_2020_2237_MOESM3_ESM.pdf]
